# Molecular-Based Nanoplatform Leads to the Formation of a Self-Indicating Responsive Drug Delivery System

**DOI:** 10.3390/molecules30081782

**Published:** 2025-04-16

**Authors:** Lingbo Zhang, Muhua Chen, Weihao Wang, Zhijie Luo, Yuhui Zheng

**Affiliations:** 1 Department of Anesthesiology, Xiangya Hospital Central South University, Changsha 410008, China; lingbo1903@outlook.com; 2 Guangzhou Key Laboratory of Analytical Chemistry for Biomedicine, School of Chemistry, South China Normal University, Guangzhou 510006, China; rareearth@126.com (M.C.); 2023022500@m.scnu.edu.cn (W.W.); 20091102@m.scnu.edu.cn (Z.L.)

**Keywords:** cellular imaging, fluorescence, nanoplatform, therapy

## Abstract

We report the design and biological evaluation of a nanoplatform featuring controllable aggregation-induced emission (AIE) behavior. The free rotation of benzene rings (4-(1,2,2-triphenylvinyl) benzaldehyde) largely suppresses fluorescence in the pure organic phase. However, water-induced molecular aggregation enhances the fluorescence signal. The delivery system follows the membrane–cytoplasm–nucleus route and it leads to apoptosis in two cancer cells (U937 cells and Hela cells). The AIE moiety accumulates in the cytoplasm, emitting a bright-blue signal, but the anticancer drug doxorubicin selectively targets the nucleus with unique red emission. The current noninvasive method with DOX-triggered apoptosis holds promise for tumor diagnosis and real-time imaging.

## 1. Introduction

Cancer chemotherapy has generated significant attention over the past few decades as tumors have become a major global healthcare problem [1]. In the strategy design of cancer chemotherapy, the drug dose needed to reach diseased regions is very important for individual patient treatment. However, conventional chemotherapeutic approaches are often associated with severe side effects and variable therapeutic responses among patients [2,3]. To address these limitations, stimuli-responsive or stimuli-degradable theranostic platforms work by integrating imaging and therapeutic functions, which is particularly interesting for controlled drug release applications [4,5,6,7]. Various external stimuli, including pH [8], light [9], heat [10], and enzymes [11], have been exploited to trigger drug release from hybrid delivery systems. Among these, pH-responsive mechanisms have drawn particular interest due to the acidic microenvironment of tumors [12].

In light of various external activations, numerous publications have focused on the careful design and preparation of drug delivery through fluorescent emission in an ordered way. The primary problem lies in the severe overlap in signals between organic dyes and drug reagents, and the loss of luminescence inside aqueous solutions is detrimental to real-time studies of therapeutic agents operated in this way. Furthermore, common organic molecules tend to aggregate together and anchor onto the surfaces of biological species, and the notorious concentration quenching effect would largely suppress the feasibility of using fluorescent sensors, especially in on-site recognition.

To overcome these shortcomings, a wide range of pH-sensitive nanoplatforms have been widely developed, exhibiting good safety performance and easy functionalization [13,14,15]. After the Tang group developed a new route of aggregate formation in a light-emitting device [16], a variety of aggregation-induced emission (AIE) materials have been prepared [17,18,19,20,21,22,23]. In recent years, AIE edifices have gained substantial attention for their dual roles in tumor sensing and therapy.

**Figure 1:**
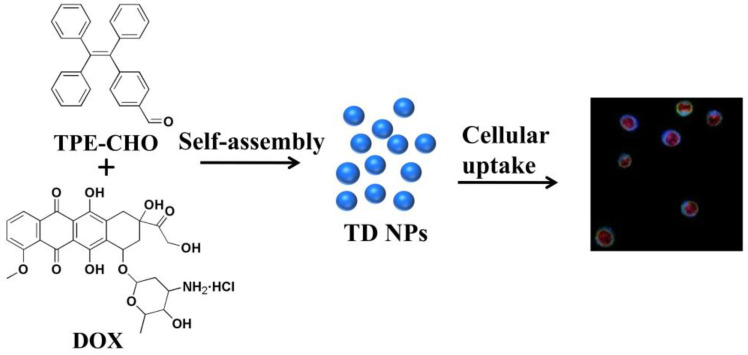
Structure of TPE-CHO and the preparation of TD NPs and the subsequent utilization of the TD NPs for cell therapy. Different color of cells indicates the co-localization of TPE-CHO with lysosomes (blue to green), the internal red fluorescence from DOX corresponds to nuclear staining.

As illustrated in Figure 1, a self-indicative nanoscale platform was constructed based on a tetraphenylethylene-bearing aldehyde group (referred as TPE-CHO). A typical cytotoxic antibiotic with anticancer features known as doxorubicin (DOX) was selected to establish the assembly framework (TD NPs) together with the above molecular-model (TPE-CHO). The routes of how the nano-assembly may penetrate cell membranes have been studied, enabling the visualization of specific intracellular targets and biological processes, thereby highlighting their potential for real-time tumor imaging and targeted therapy.

## 2. Results and Discussion

**Figure 2:**
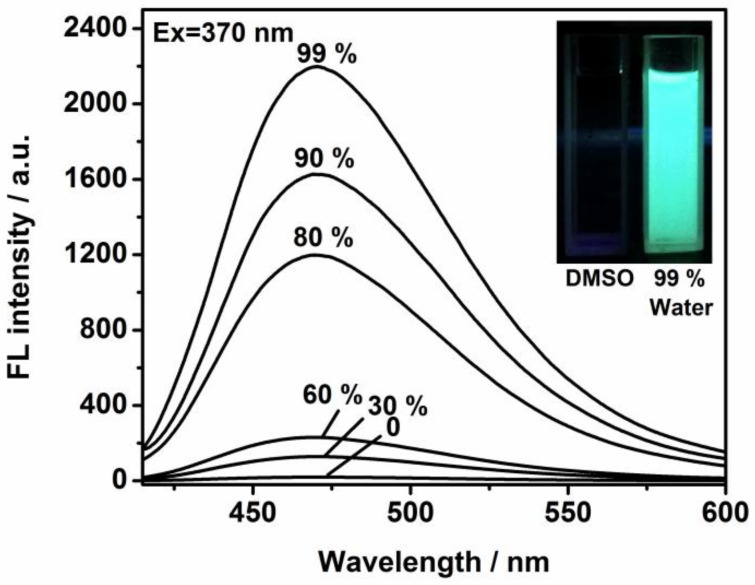
Emission changes of TPE-CHO (25 μM) in DMSO/water mixtures with different concentrations of water.

The design and synthesis of the molecular-based motif are illustrated in Figure 1, with particular emphasis on the self-assembly process. The functional framework was achieved in relatively high yield. Bromotriphenylethylene was treated with 4-formylphenylboronic acid in dried toluene to afford the intermediate compound. Then, it was reacted with tetrakis (triphenylphosphine) palladium in argon to generate 4-(1,2,2-triphenylvinyl) benzaldehyde. To evaluate its potential for cellular imaging and biological applications, we explored its photophysical behavior in mixed aqueous–organic solvents. The evolution curves in the emission spectra were tracked via the titration studies. The intrinsic excitation and emission bands were located at 370 and 490 nm (Figure S1). As provided in Figure 2, the free ligand in DMSO solution possessed a very weak fluorescence signal in terms of fast exchange and rotation within molecules. But the step-by-step incorporation of water totally changed the emission profiles and efficiency. After the addition of 99% of water, a sky-blue emission peak with high intensity was monitored and the band was enormously increased to more than 80 times of its original value (Figure 2). This switched-on change was in agreement with the detection by naked eye (from weak emission to bright blue) of the solutions due to irradiation by the ultra-violet lamp (inset of Figure 2). The well-established molecular model with unique luminescence was chosen as an effective host for immobilizing diverse anticancer drug species such as DOX. The nanoplatform’s internal structure, bearing positive charge and extended aromatic systems, facilitates the encapsulation of drug molecules via dynamic covalent bond interaction and π-π stacking forces.

Upon irradiation at 370 nm, a striking change was obtained and the emission band situated at 470 nm was varied in the presence of DOX at different concentrations (Figure S2). The increasing loading of drugs resulted in the progressive quenching of TPE-CHO fluorescence, suggesting an interaction between the host and drug molecules. This emission change is attributed to a Forster resonance energy transfer (FRET) process. The intrinsic emission feature of DOX (λ_ex_ = 490 nm, λ_em_ = 595 nm) is given in Figure S3. To fully verify the possibility of such a mechanism, the emission of the aggregated structure and the absorption of DOX were measured (Figure S4). Spectral overlap was observed between the two species and the transfer efficiency was high. In addition, the full spectral range of DOX was studied and its emission band could be extended to 595 nm, which could be distinguished from the blue signal of the host (Figure S3). In this way, we could understand that the individual DOX molecule possesses red emission, but that the interpenetration effect between DOX and TPE-CHO induced the changes. In Figure S5, the peaks at 3045 cm^−1^ and 3017 cm^−1^ were ascribed to the unsaturated C-H stretching vibration. The signals at 2815 cm^−1^ and 2719 cm^−1^ were caused by the C-H stretching vibration in aldehyde groups (Figure S5). However, the aldehyde signal vanished in TD NPs, indicating that the aldehyde and amino moiety in DOX react to yield the Schiff base. Therefore, the assembly-caused quenching could be correlated to the Forster resonance energy transfer (FRET) process. When the pH value varied during the cellular uptake process, the formation of nanoparticles decomposed since the C=N linkage is vulnerable to the acid-catalyzed hydrolysis reaction.

**Figure 3:**
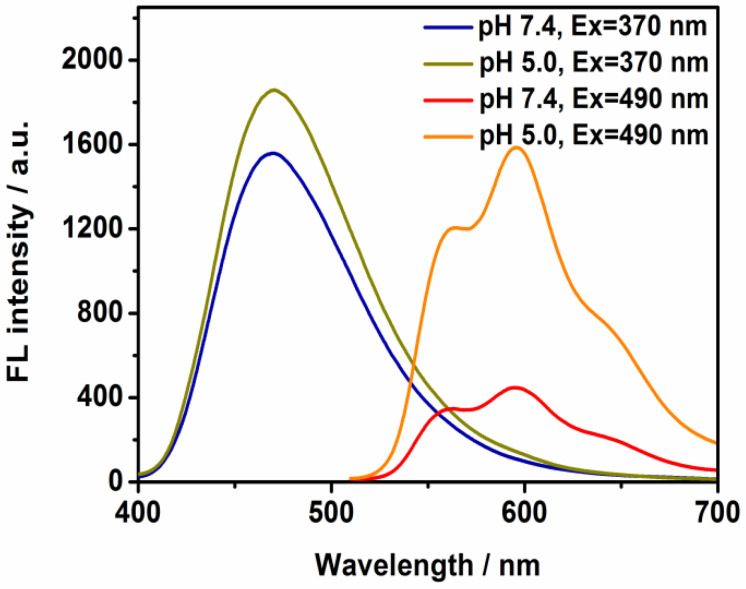
Fluorescence spectra of TD NPs (25 μM) with different excitation wavelengths (Ex = 370 nm and 490 nm) at pH 7.4 and 5.0.

A powerful approach that includes the luminescence change and release of therapeutic agent simultaneously would be extremely important in biomedical applications. Previous studies have shown that drug release is enhanced at mildly acidic pH, improving therapeutic efficacy [23]. In this work, the controllable process of the nanostructure was indicated by observing the change in the assembly under pH-dependent conditions. In the presence of DOX (Figure 3), a clear increase in the red emission was measured at pH = 5.0. These sharp changes verified that the majority of drug molecules would be anchored to the tumor targets based on the lower pH environment and that the therapy efficiency could be reinforced. The time-duration experiments were also performed in the presence of acidic environment (pH = 5); it was found that the TD NPs possessed pH sensitivity and that the nanodomain enabled the visualization of signal change by recording the increasing emission intensity (Figure S6). The peak intensity was almost saturated after 60 min.

**Figure 4:**
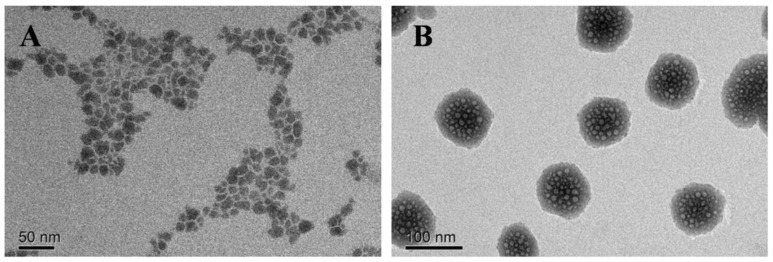
TEM images of TPE-CHO NPs (**A**) and TD NPs (**B**) (pH = 7.4).

The internal morphology of the organic scaffold and the aggregated species was examined by TEM images, as shown in Figure 4. The dispersion of independent TPE-CHO granules could be visualized and the average size was found to be ~30 nm. Since the DOX moiety was immobilized, the individual particles were observed to aggregate into large clusters and the regular spheres were measured to be around 100 nm (Figure 4). The dimensions of the loosely assembled nanostructure might be in the suitable size range for therapy purposes and may be capable of cellular penetration [24]. These size evolution changes due to the introduction of DOX molecules demonstrate that the charge attraction and π-π stacking led to the aggregation of the two species.

The size evolution upon DOX encapsulation is consistent with previous studies reporting drug-induced aggregation in aqueous environments [25,26]. DOX frequently possesses the drawback of reduced accumulation in living cells since it is a host for over-expressed cancer cells, causing an inhibited anti-tumor effect. On the other hand, the excessive use of DOX may lead to toxicity to a variety of organs. Therefore, it is necessary to encapsulate it into a confined space to improve its quality, efficacy, and safety. A low-molecular-weight molecule may be compatible to avoid toxicity. Its surface group could efficiently assist in the adsorption of chemotherapeutic drug species and dynamic chemical bonding would be established. The stabilization of nanoscale delivery models can be realized via the entrapment the drug in terms of reactive functional terminals, such as amino and aldehyde groups in DOX and TPE-CHO [27]. We have to mention that there was no direct relationship between the luminescence change and the micro-structure. Although the nanoparticle might release proportions of DOX at slightly lower pH values, the emission band varied greatly, as indicated by Figure 3. However, the apparent size and shape of the nanoparticle were relatively stable at pH = 5 (Figure S7). This observation suggests that the nanoplatform maintains structural integrity during partial drug release, which could be beneficial for sustained therapeutic action in acidic tumor microenvironments.

**Figure 5:**
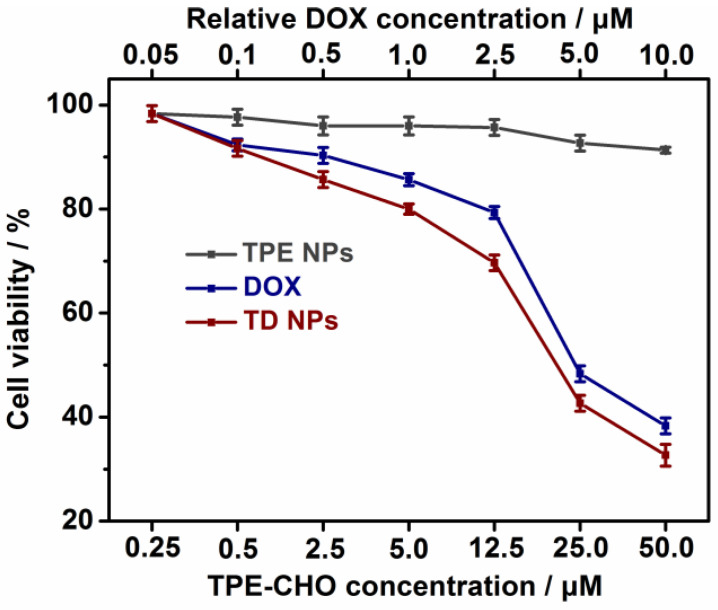
Cell viability of TPE-CHO NPs, DOX, and TD NPs towards to U937 cells.

**Figure 6:**
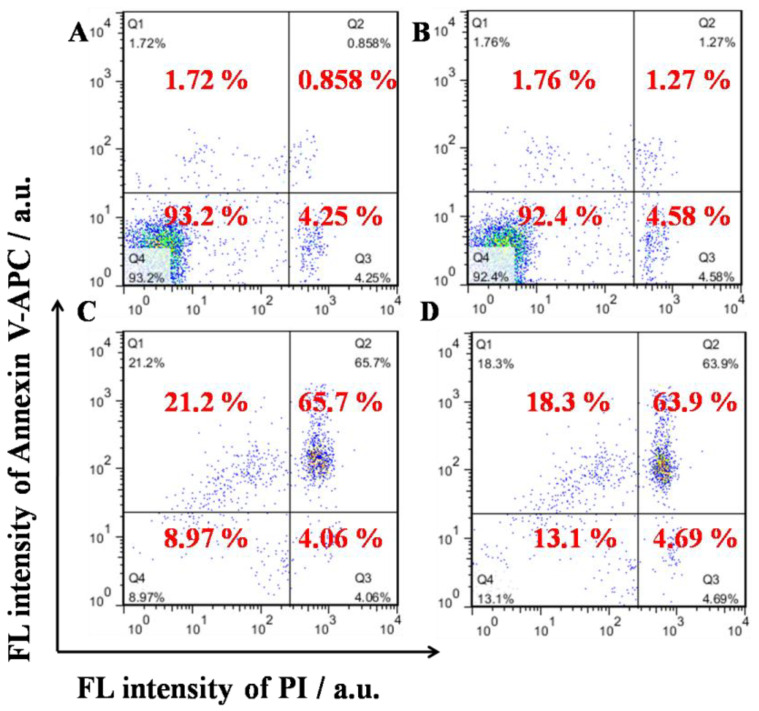
Representative distribution of U937 (**A**,**C**) and Hela cells (**B**,**D**) obtained by Annexin V-APC/PI staining experiments. Cells of (**A**,**B**) were treated with 25 μM TPE-CHO NPs, and (**C**,**D**) were treated with 25 μM TD NPs. Color dots from blue to red refer to cell density increase.

The cytotoxicity of TPE-CHO, free DOX, and TD NPs was carefully assessed in two cell systems (U937 and Hela cells) by the MTS method, as given in and Figure 5 and Figure S8. It was observed that 24 h of incubation with TPE-CHO triggered cell viabilities higher than 90%, indicating that the synthesized organic framework possessed enough biocompatibility during in vitro experiments. After the treatment with DOX and TD NPs, a sharp decrease in the cell viability rate was obtained. Moreover, the viability of cells loaded with TD NPs was clearly less than that of pure DOX. The results reveal that a more effective anticancer drug delivery rate can be realized by the nanostructure. Additionally, the specific apoptosis process based on different components was studied by flow cytometry (Figure 6). U937 cells were loaded with TPE-CHO at 25 μM and high viability was observed. The incubation with TD NPs gave rise to completely different results in later apoptosis rates and dead cells. These values (65.7% and 21.2%) were much larger than for the pure TPE-CHO sample and the data are consistent with the above measurement of tumor cell viability.

Release behavior was demonstrated by carrying out the experiment at pH = 5, and Figure S9 shows the drug release profile. As for the appended doxorubicin, its release rate was grew quickly, with around 82% at pH = 5 within 60 min. This change can be ascribed to the protonation and following dissociation of the Schiff base by way of hydrolysis, causing effective DOX release. The process leads to the extraction of DOX from TD NPs, again providing valuable insights that support the emission change in Figure 3.

**Figure 7:**
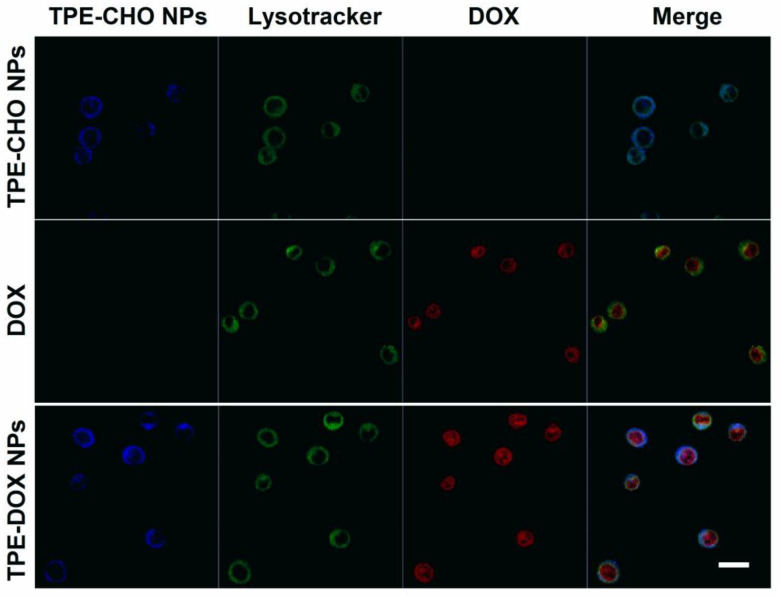
Confocal microscopy images of U937 cells cultured with TD NPs (25 μM), free DOX (5 μM), and TD NPs (25 μM) and co-stained with Lysotracker Green after a 4 h incubation. Different color of cells indicates the co-localization of TPE-CHO with lysosomes (blue to green), the internal red fluorescence from DOX corresponds to nuclear staining.

With the aim of verifying the practical potential of TD NPs as a functional marker for live cells imaging, U937 cells were incubated with TPE-CHO molecules (25 μM) under physiological conditions. Confocal microscope images revealed bright-blue luminescence derived from the fluorescent signal by organic edifice, indicating that a variety of aggregated molecules travelled across the cellular membrane (Figure 7). After sole incubation by the DOX molecule, the intracellular distribution of free DOX in U937 cells was also observed. In a different way, very intense blue fluorescence was captured in the cell cytoplasm due to the addition of TD NPs for four hours. The cellular uptake of the synthesized nano-assembly supports the feasibility of its intracellular transport. Furthermore, a red color was identified in the nucleoplasm of the cells loaded with TD NPs and the cellular imaging features were in sharp contrast with the sample treated with TPE-CHO (Figure 7). The human tissue cell lymphoma cells’ exposure to the new delivery system could be tracked via the membrane-to-nuclear transport method. The adaptable nanostructure released DOX under a mild acidic environment and migrated into the cell nucleus.

**Figure 8:**
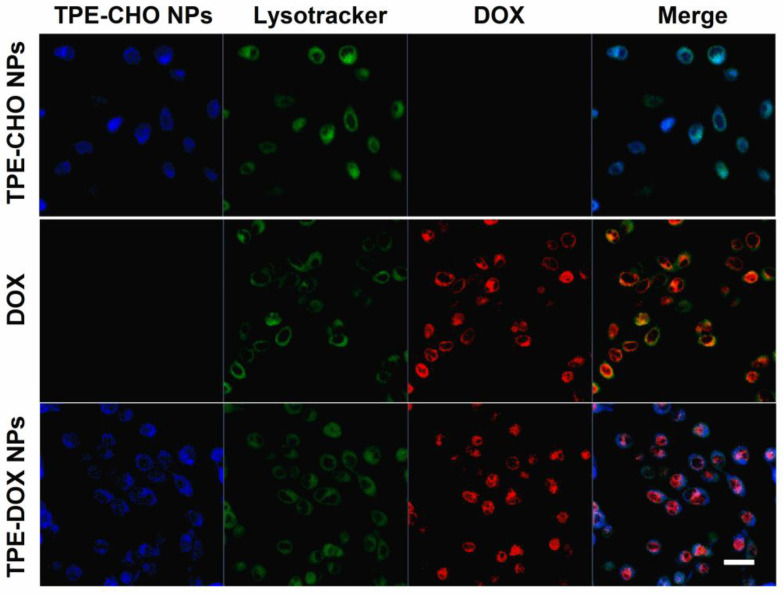
Confocal microscopy images of Hela cells cultured with TD NPs (25 μM), free DOX (5 μM), and TD NPs (25 μM) and co-stained with Lysotracker Green after a 4 h incubation. Different color of cells indicates the co-localization of TPE-CHO with lysosomes (blue to green), the internal red fluorescence from DOX corresponds to nuclear staining.

Lysotracker Green staining further supported these findings by demonstrating the co-localization of TPE-CHO with lysosomes, whereas the red fluorescence from DOX corresponded with nuclear staining. This behavior is consistent with the known acidic pH of lysosomes and tumor cell compartments, which promotes the acid-triggered disassembly of TD NPs through hydrolysis of the imine (C=N) bond. Accordingly, an anticancer drug could be released from the nano-carrier and penetrated into tumor cells at a lower pH, and the therapy effects would be enhanced. The performance of TD-NPs as a functional prodrug in Hela cells was also explored. These adherent cell lines were stained with TPE-CHO, DOX, and TD NPs (Figure 8). The very distinguished blue luminescence was observed in the cytoplasm and the red signal was densely distributed in nuclei throughout the membrane during the nuclear process. In this sense, in vitro imaging profiles were recorded. The results indicate that TD NPs could be employed for specific drug delivery, and the real-time observation of fluorescence evolution in cellular systems would also be possible.

## 3. Methods and Materials

### 3.1. Materials and Apparatus

4-Formylphenylboronic acid, Bromotriphenylethylene, Tetrabutylammonium bromide (TBAB), doxorubicin hydrochloride (DOX), Dulbecco’s modified medium (DMEM), RPMI1640 medium, fetal bovine serum (FBS), antibiotic solution (penicillin and streptomycin), and 0.25% trypsin-EDTA were obtained from Sigma-Aldrich (St. Louis, MO, USA). The CellTiter 96 AQueous One Solution Reagent (MTS kit) was obtained from Promega (Madison, WI, USA). Lysotracker Green and an Annexin V-APC/Propidium Iodide (PI) kit were purchased from BD Biosciences (Franklin Lakes, NJ, USA). All the other chemicals were analytical-grade reagents and were used without further purification.

Fluorescence spectra were recorded using a Hitachi F-4600 fluorescence spectrophotometer (Tokyo, Japan) with a xenon lamp as the light source. The excitation slit width was 5 nm and the emission slit width was 5 nm (scan speed, 1200 nm/min; voltage, 700 V). Confocal images were captured with a Zeiss confocal laser scanning microscope (LSM710, Jena, Germany) equipped with a laser at 405 nm. MTS was measured at 490 nm using a Polarstar microplate (BMG Labtech, Offenburg, Germany). Cell apoptosis analysis was performed on a BD Accuri C6 Flow Cytometer (BD Biosciences).

### 3.2. Synthesis of 4-(1,2,2-Triphenylvinyl) Benzaldehyde (TPE-CHO)

4-Formylphenylboronic acid (0.75 g, 5 mmol) and Bromotriphenylethylene (1.116 g, 3.4 mmol) were dissolved in a mixture of toluene (20 mL), TBAB (0.107 g, 0.34 mmol), and 2 M potassium carbonate aqueous solution (6 mL). After degassing and purging with Ar gas, Pd (PPh_3_)_4_ (0.033 g, 2.90 × 10^−3^ mmol) was added. The reaction mixture was refluxed at 90 °C for 24 h. After that, the mixture was poured into water to quench the reaction, and the product was extracted three times with ethyl acetate. The organic layer was dried over anhydrous sodium sulfate and concentrated. The crude product was purified by column chromatography using n-hexane/CH_2_Cl_2_ (2:1 by volume) as the eluent to give the target compound, a light yellow solid. ^1^H NMR spectra were measured by a Bruker ultrashield 600 equipped with a dedicated 5 mm proton probe on a 0.5 mL sample, and all chemical shifts were referred to an internal reference. ^1^H NMR: (600 MHz, CDCl3, δ (ppm)): 9.90 (s, 1H), 7.62 (d, 2H), 7.21 (d, 2H), 7.13–7.00 (m, 15H).

### 3.3. Preparation of TD NPs

TD NPs were prepared by the following method. TPE-CHO (36 mg, 0.1 mmol) and DOX (11.6 mg, 0.02 mmol) were dissolved in 5 mL DMSO and underwent ultra-sonication at room temperature for 10 min to obtain a transparent mixture solution. Afterwards, 50 μL of PBS buffer solution was added to the mixture and the pH value was adjusted to 7.4 with the addition of 0.1 M NaOH solution. The mixture was stirred at room temperature for 1 h. After that, the above mixture was added dropwise into PBS buffer solution (pH 7.4) under ultra-sonication, and TD NPs were obtained after 5 min. Similarly, TPE-CHO NPs were prepared by first adding TPE-CHO in DMSO dropwise into PBS buffer solution (pH 7.4) under ultra-sonication. UV-vis absorption spectra were collected by using a UV 2500 spectrophotometer (Techcomp, Shanghai, China) with a wavelength covering from 200 to 650 nm at room temperature, within 10 mm path-length quartz cells. Transmission electron microscopic images were measured by a JEOL JEM-2100 UHR microscope (Akishima, Japan) under an acceleration voltage of 200 kV, and the nanoparticles were dispersed over the TEM sample grid; the image acquisition time per frame was set as 3 s.

### 3.4. The Encapsulation Efficiency and DOX Release Experiment

Based on the measurement of the absorption band (480 nm) of DOX standard samples at different concentrations, a linear equation between absorbance and DOX concentration was achieved (y = 0.0059x + 0.01925, R^2^ = 0.99), and the relevant concentrations could be determined by using the standard curve. Its encapsulation efficiency in TD NPs was calculated to be 17% (efficiency = (Total amount of DOX added − free DOX)/Weight of TD NPs). As for the release experiment, the release curve was obtained by using a dialysis approach. TD NPs were dispersed and placed within a dialysis bag and the sample was immersed at pH = 5. The cumulative release of DOX at diverse time intervals was recorded and the amount of released DOX (out of the semipermeable dialysis bag) was quantified by ultraviolet spectroscopy, with absorption at 480 nm.

### 3.5. Cell Lines and Cell Culture

U937 cells (human histiocytic lymphoma cells) and Hela cells (human cervical cancer cells) were cultured in RPMI 1640 medium and DMEM medium, respectively, both supplemented with 10% heat-inactivated fetal bovine serum (FBS) and 1% antibiotics penicillin (PS) and maintained at 37 °C under humidified conditions of 95% air and 5% CO_2_.

### 3.6. MTS Assay

The cytotoxicity of TPE-CHO NPs, DOX, and TD NPs on U937 cells and Hela cells was assessed with MTS kits. U937 and Hela cells were grown on 96-well microplates at a density of 5000 cells/well and incubated at 37 °C with 5% CO_2_ for 24 h. Among them, 8 wells were filled culture medium only as a control group. After removal of the medium, the cells were incubated for another 24 h with fresh medium containing different concentrations of TPE-CHO NPs, DOX, or TD NPs. Thereafter, 20 μL of MTS solution was added to each well and incubated for another 2 h, and the absorbance at 490 nm was collected by using a microplate reader for further analysis.

### 3.7. Cell Apoptosis Analysis

Cell apoptosis of TPE-CHO NPs and TD NPs was assessed by flow cytometry. U937 and Hela cells were grown on 96-well microplates at a density of 5 × 10^5^ cells/well at 37 °C with 5% CO_2_ for 24 h, respectively. Then, the original culture medium was replaced with fresh medium containing TPE-CHO NPs or TD NPs (25 µM). The samples were incubated for another 24 h. Thereafter, the cells were washed with binding buffer twice, centrifuged, and another 100 μL binding buffer was added. Finally, 3 μL of Annexin V-PE and PI were added to each sample. The samples were further incubated for 15 min and analyzed by flow cytometry.

### 3.8. Cell Imaging

For cell imaging, U937 and Hela cells were seeded on Lab-Tek chambered cover glass (4-well, Thermo Scientific, Waltham, MA, USA) at a density of 5 × 10^4^ cells/well and grown to 70% confluency. The cells were washed with culture medium and incubated for another 4 h in the fresh medium containing TPE-CHO NPs (25 μM), DOX (5 μM), or TD NPs (25 μM). Subsequently, the cells were washed three times with PBS and then contained in 200 nM Lysotracker Green for 20 min. Imaging was performed using a Zeiss confocal laser scanning microscope (LSM710, Jena, Germany).

## 4. Conclusions

With the ongoing advancement of nanoscale delivery systems, the development of structurally verified and functionality effective drug carriers remains essential. In this work, the employment of aggregation-induced emission molecules demonstrated unique features compared with routine fluorophores. According to the results, TPE-CHO possessed relatively low emission signals in pure DMSO. The addition of water caused an 80-fold increase in the emission profiles. The DOX-appended nanostructure (TD NPs) could travel through two kinds of cancer cells (including U937 cells and Hela cells). Its strong affinity in cellular systems supports this AIE-based assembly’s value in cellular imaging and anti-tumor drug carriers. These results highlight the potential of AIE-related frameworks for real-time drug tracking and the further development of molecular-based models in clinical applications.

## Data Availability

Data is contained within the article and Supplementary Materials.

## Supplementary Materials

The following supporting information can be downloaded at: https://www.mdpi.com/article/10.3390/molecules30081782/s1, Figure S1: Excitation and emission spectra of TPE-CHO NPs (25 μM). Figure S2: Emission changes of TPE-CHO with different molar ratios of DOX. Figure S3: Excitation and emission spectra of DOX (5 μM). Figure S4: Emission and UV-Vis spectra of TPE-CHO NPs and DOX. Figure S5: Infrared spectra of TD NPs, TPE-CHO NPs and DOX. Figure S6: Time-dependent changes in the emission intensity at pH = 5. Figure S7: TEM image of TD NPs at pH = 5 after1 h. Figure S8: Cell viability of TPE-CHO NPs, Dox and TD NPs towards to Hela cells. Figure S9: Time duration responsive release curve at pH = 5.
