# Supplementary material for: Molecular-Based Nanoplatform Leads to the Formation of a Self-Indicating Responsive Drug Delivery System
Source: Molecules. 2025 Apr 16;30(8):1782. doi: 10.3390/molecules30081782 (PMC12029280; doi:10.3390/molecules30081782)
Supplement: Supplementary file 1 [file molecules-30-01782-s001.zip › molecules-3563038-supplementary.pdf]

Supplementary Materials

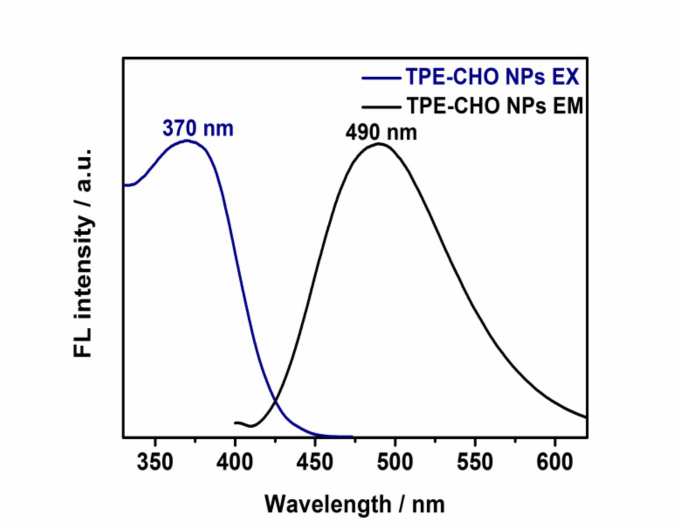

Figure S1. Excitation and emission spectra of TPE-CHO NPs (25 μM).

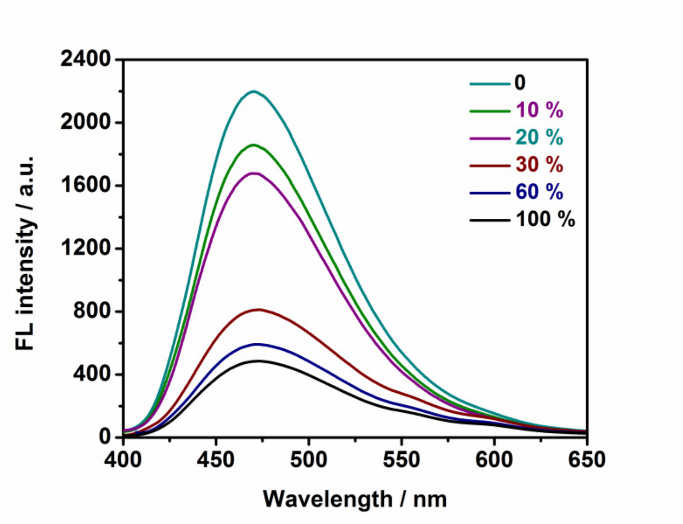

Figure S2. Emission changes of TPE-CHO with different molar ratios of DOX.

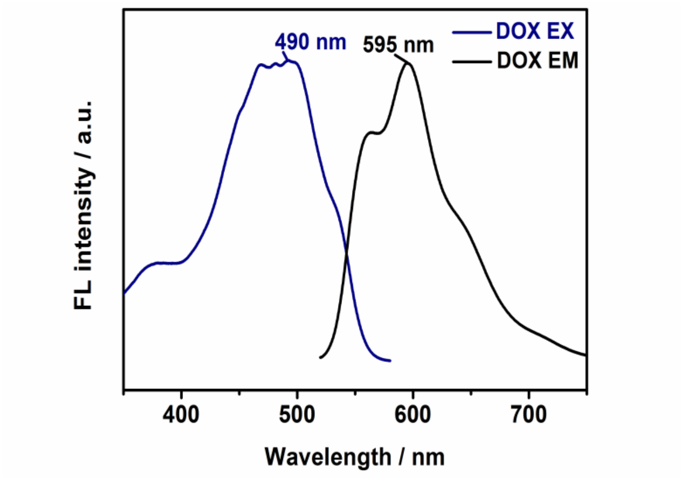

Figure S3. Excitation and emission spectra of DOX(5 μM).

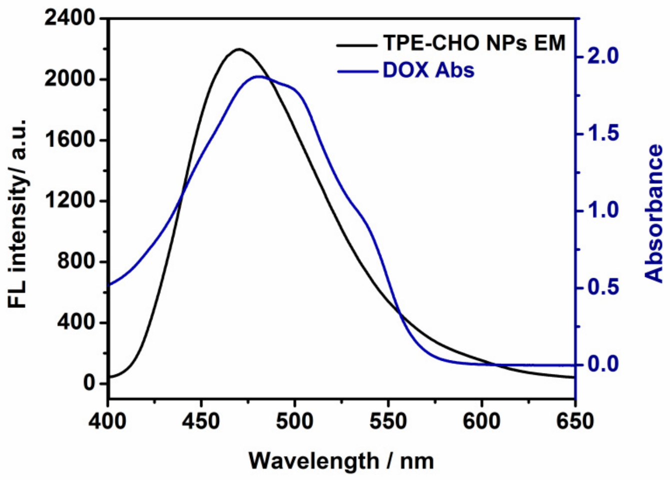

Figure S4. Emission and UV-Vis spectra of TPE-CHO NPs and DOX respectively.

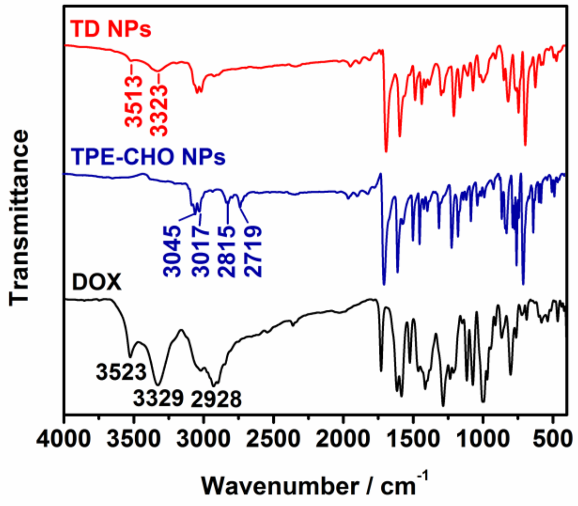

Figure S5. Infrared spectra of TD NPs, TPE-CHO NPs and DOX.

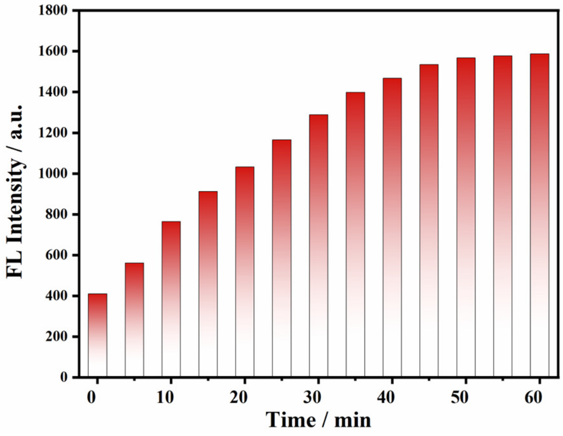

Figure S6. Time-dependent changes in the emission intensity at pH = 5.

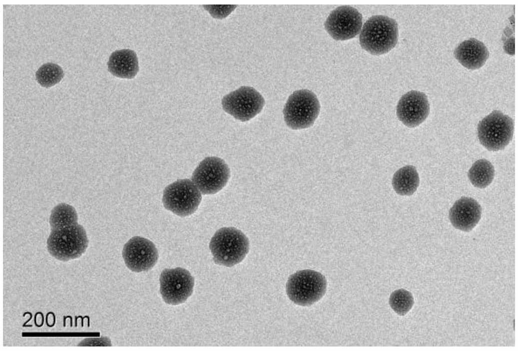

Figure S7. TEM image of TD NPs at pH = 5 after1 hour.

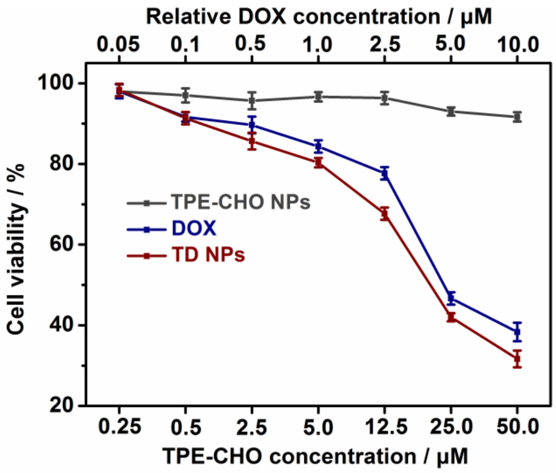

Figure S8. Cell viability of TPE-CHO NPs, Dox and TD NPs towards to Hela cells.

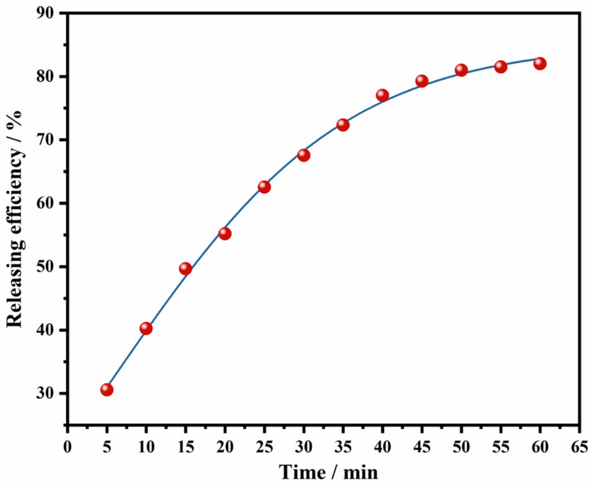

Figure S9. Time duration responsive release curve at pH = 5.
